# Oxidative Stress: A Possible Trigger for Pelvic Organ Prolapse

**DOI:** 10.1155/2020/3791934

**Published:** 2020-09-01

**Authors:** Radu Dragos Marcu, Dan Liviu Dorel Mischianu, Lucian Iorga, Camelia Cristina Diaconu, Mihaela Surcel, Adriana Narcisa Munteanu, Carolina Constantin, Gheorghita Isvoranu, Ovidiu Gabriel Bratu

**Affiliations:** ^1^Clinic of Urology, University Emergency Central Military Hospital, 134 Calea Plevnei, 010825 Bucharest, Romania]; ^2^University of Medicine and Pharmacy, 37 Dionisie Lupu Street, 020021 Bucharest, Romania; ^3^Academy of Romanian Scientists, 54 Spl. Independentei, 050094 Bucharest, Romania; ^4^Department of Internal Medicine, Clinical Emergency Hospital of Bucharest, Bucharest, Romania; ^5^Immunology Department, National Institute of Pathology, 99-101 Spl. Independentei, 050096 Bucharest, Romania; ^6^Animal Husbandry, National Institute of Pathology, 99-101 Spl. Independentei, 050096 Bucharest, Romania

## Abstract

Pelvic organ prolapse is a frequent health problem in women, encountered worldwide, its physiopathology being still incompletely understood. The integrity of the pelvic-supportive structures is a key element that prevents the prolapse of the pelvic organs. Numerous researchers have underlined the role of connective tissue molecular changes in the pathogenesis of pelvic organ prolapse and have raised the attention upon oxidative stress as an important element involved in its appearance. The advancements made over the years in terms of molecular biology have allowed researchers to investigate how the constituent elements of the pelvic-supportive structures react in conditions of oxidative stress. The purpose of this paper is to underline the importance of oxidative stress in the pathogenesis of pelvic organ prolapse, as well as to highlight the main oxidative stress molecular changes that appear at the level of the pelvic-supportive structures. Sustained mechanical stress is proven to be a key factor in the appearance of pelvic organ prolapse, correlating with increased levels of free radicals production and mitochondrial-induced fibroblasts apoptosis, the rate of cellular apoptosis depending on the intensity of the mechanical stress, and the period of time the mechanical stress is applied. Oxidative stress hinders normal cellular signaling pathways, as well as different important cellular components like proteins, lipids, and cellular DNA, therefore significantly interfering with the process of collagen and elastin synthesis.

## 1. Introduction

Pelvic organ prolapse (POP) is one of the most common female pathologies, encountered especially in patients over 50 years old. Its incidence increases with age, being more frequent in older patients compared to young and middle age females, and also with the number of vaginal births [1–3]. There are numerous factors that seem to favor POP pathogenesis: obesity, chronic constipation, sustained physical activities that increase the intra-abdominal pressure, menopause-associated hormonal changes, collagen disorders, history of pelvic surgery, and radiotherapy. POP-associated symptoms can significantly impact the patient's life style, usually leading to anxiety, depression, self-isolation, and low self-esteem, as well as to a significant decline in terms of sexual intercourse frequency (especially in younger and active patients) [4, 5].

The support of the pelvic organs is ensured by the levator ani muscles, pelvic fascias, and ligaments. The dysfunctions of these structures have been shown to alter their supportive role, thus favoring the descent of the pelvic organs and other associated pathologies and symptoms [6]. The supportive effect of these structures depends not only on the quantity of their cellular components but also on their quality [7]. Numerous studies have underlined the role of connective tissue molecular changes in the pathogenesis of pelvic organ prolapse.

The pelvic connective tissue consists of fibrous elements like collagen and elastin, which are the principal components of the extracellular matrix (ECM) and also of the elastogenic cells, represented by smooth muscles cells and fibroblasts [8]. Other important components of the ECM are fibronectin, fibrinogen, vitronectin, laminins, thrombospondin, integrins, and other glycoproteins involved in the cellular adherence process, which is mediated by the integrin membrane receptors [9]. Integrins are indispensable for the ECM proteins metabolism, as well as for the cellular turnover, this being a constant process [10, 11].

## 2. Collagen and Elastin

Collagen is the main component of the connective tissue, accounting for up to 70-80% [12]. There have been identified 29 types of proteins that can be included in the collagen family, but the most representative ones for the pelvic connective tissues are type I and III collagens, these two collagen types being responsible for the normal integrity and functionality of the pelvic floor supportive structures [13]. Collagen precursors known as procollagen are released by fibroblasts into the extracellular space, where they become tropocollagen under the action of collagen peptidases that remove the ends of this molecule. Under the action of lysyl oxidase, the tropocollagen molecules bind together and form the collagen fibril [14].

Type I collagen is known for its high stretching ability and for the fact that it has a major role in strengthening the pelvic structures, whereas type III collagen, being a much smaller protein compared with type I collagen, is responsible for tissue flexibility, along with elastin [15]. The percentage of collagen within these pelvic connective tissues, as well as the ratio between type I and III collagens, are important elements that significantly contribute to the integrity of the pelvic supporting structures. Therefore, collagen dysfunction or the existence of an abnormal ratio between type I and type III collagens will eventually disrupt the strength of the pelvic tissues, as well as their elasticity and relaxation ability [16].

Two important enzymes modulate the collagen metabolism and the ECM turnover: matrix metalloproteinases (MMPs) and tissue inhibitors of matrix metalloproteinases (TIMP). MMPs are a group of proteolytic enzymes that degrade all ECM proteins. MMPs belong to the ECM protein family, but they can also be found within cells, especially MMPs 1, 2, and 11 [17]. MMPs present three domains: a propeptide domain, a catalytic domain (the site of the zinc ion), and the hemopexin domain, which is responsible for the interactions of different types of MMPs, as well as with TIMPs. They are secreted as inactive enzymes, known as zymogens; their activation is calcium and zinc dependent, and it implies the cleavage of the propeptide domain [18]. MMP gene expression has been identified in fibroblasts, endothelial cells, macrophages, monocytes, and neutrophils. MMP concentrations are usually constant, this process being mediated by hormones, cytokines, growth factors, ultraviolet radiation, and cellular interaction. MMP expression is usually enhanced by cytokines like tumor necrosis factor- (TNF-) *α* and interleukin (IL) 1 and 6, as well as by growth factors like transforming growth factor- (TGF-) *β*, platelet-derived growth factor (PDGF), fibroblast growth factor (FGF), and epidermal growth factor (EGF). IL-4, corticosteroids, heparin, and retinoic acid have been demonstrated to inhibit the expression of MMPs [14, 19].

Over the time, there have been identified more than 20 MMPs (23 in humans) [20] that can be classified as follows: collagenases (MMPs 1, 8, 13, and 18), gelatinases (MMPs 2 and 9), stromelysins (MMPs 3, 10, 11, and occasionally MMP 19, referred as stromelysin-4), membrane-type MMP (MMPs 14, 15, 16, 17, 24, and 25), matrilysin (MMPs 7 and 26), and other MMPs (MMP 12-metalloesterase) [21, 22].

TIMP counterbalances the action of MMP. TIMPs bind with MMPs (both with the inactive MMPs—zymogens and also with the active MMPs) and form a complex that inhibits the activation of MMPs, the connection of MMPs with their substrates and their activity, therefore significantly reducing the collagen degradation process and the destruction of other ECM proteins [17]. As in the case of MMPs, TIPM expression is also modulated by a number of cytokines and growth factors. Several POP studies that have analyzed MMPs and TIMPs as cellular markers for POP have identified high MMP levels and/or lower TIMP concentrations, underlining that an imbalance between MMPs and TIMPs can severely alter the quality of the pelvic connective tissues, thus favoring pelvic organ prolapse pathogenesis [23, 24]. It has been reported that POP patients do not have inferior collagen concentrations at the level of the pelvic-supportive structures compared to the non-POP patients and that the molecular mechanism involved in POP pathogenesis is in fact an accelerated process of collagen degradation that exceeds its synthesis [25].

Elastic fibers are other important components of the pelvic-supportive structures, due to the fact that they confer high stretching and passive recoil capabilities to the pelvic tissues. Like collagen, these elastic fibers are major components of the ECM, and they present a complex molecular biology. Elastin and microfibrils are the main components of elastic fibers, elastin accounting for up to 90% of their mass [26]. Elastin synthesis occurs during the last months of the intrauterine life, as well as in the first months after birth, afterwards this process being negligible. There are several pathological situations that are accompanied by new elastin synthesis, but its quality has proven to be inferior to that of the one produced during its normal synthesis period [27, 28]. Microfibrils are fibrillar elements that are characterized by high concentrations of glycoproteins like fibulins (FBLNs), fibrilins (FBNs), microfibril-associated glycoproteins (MAGPs), elastin-binding proteins, glycosaminoglycans, and lysyl oxidase [14].

Elastin synthesis is a complex process that originates at the level of the elastogenic cells, and it implies several precise steps. Tropoelastin (TE), a monomeric precursor of elastin, is secreted by smooth muscle cells and fibroblasts. TE monomers associate with each other and form lager polymers that will be deposited on the microfibrils surface. The polymerization process is mediated by several proteins located at the interface of elastin and microfibrils: FBLNs 1, 2, 4, and 5, FBNs 1 and 2, MAGPs, lysyl oxidase (LOX), and LOX-like proteins (LOXL) [26, 29, 30]. Initially, TE monomers interact and form small aggregates. FBLNs 4 and 5 favor the interaction between these small aggregates and therefore their assembly into larger polymers, as well as their microfibrillar deposition. Once deposited on the microfibrils surface, several microfibrillar elements (FBNs 1 and 2 and MAGPs) realign the TE polymers, thus favoring a new cross-linking process that will result in mature elastic fibers [31, 32]. FBLN 5 is an essential component of this process, without which polymerization would not be possible. Acting as a ligand for different integrins located on the cellular surface (avb3, avb5, and a9b1), FBLN 5 facilitates the connection between those cells and elastic fibers [10, 11]. Animal studies conducted on *Fbln5^−/−^* mice have demonstrated that FBLN 5 deficiency is associated with pelvic organ prolapse [33].

An important step of this polymerization process is catalyzed by LOX [26]. Several studies have investigated the role of LOX in normal elastogenesis, as well as LOX-associated anomalies and pelvic organ prolapse. LOX and LOX-like proteins 1-4 are encoded by five genes; therefore, disturbances of the encoding process or any other problems in terms normal LOX or LOXL proteins synthesis could lead to an altered elastogenesis [29]. Clinical research has demonstrated that deficient LOXL1 mice associate a higher risk of developing POP [30].

Considering the fact that TE is made out of several amino acids (lysine, proline, cysteine, threonine, arginine, tyrosine, leucine, methionine, phenylalanine, and valine) that are sensible to oxidative stress, it is safe to say that any oxidative stress- (OS-) induced changes at this level could alter the elastogenesis process by providing low-quality elastic fibers [14, 34].

## 3. Mitochondria, Krebs Cycle, and Oxidative Stress

In recent years, the interest regarding the role of OS in pelvic organ prolapse pathogenesis has increased. The advancements made over the years in terms of molecular biology have allowed researchers to investigate how the constituent elements of the pelvic-supportive structures react in conditions of OS. The majority of the existing studies that have focused on this subject have concluded that OS is a key element of POP pathogenesis due to the fact that it disturbs cellular homeostasis. OS hinders normal cellular signaling pathways, as well as different important cellular components like proteins, lipids, and cellular DNA; therefore, it interferes with the process of collagen and elastin synthesis, significantly altering it [35–37].

OS denotes the existence of an imbalance between oxidative agents and antioxidants that can neutralize them. In normal conditions, the production of free radicals is counterbalanced by the antioxidants activity, OS being negligible [38]. Furthermore, free radicals act as messengers in several cellular signaling pathways, being involved in gene expression, protein phosphorylation, activation of several cellular receptors and transcriptional factors, cellular differentiation, immune response, and apoptosis, thus regulating cellular activity and cellular growth [39, 40]. Nevertheless, a disruption of the normal free radical homeostasis associated with their increased production will unavoidable damage vital cellular components, as we have previously mentioned.

Free radicals are the result of an increased cellular metabolic activity known as endogenous production (infections, inflammatory and/or immune system response to different agents, malignancies, ischemia, and physical and mental stress), as well as of several exogenous factors like smoking, irradiation, alcohol abuse, drugs (bleomycin, cyclosporine, and gentamicin), solvents, herbicides, diabetes, and heavy metals (iron, cadmium, lead, mercury, and arsenic) [41–43].

The principal source for production of free radicals is the mitochondria, but they are also produced by inflammatory and endothelial cells, during reactions involved in the respiratory chain, the cytochrome P450 system, and phagocytosis or during the synthesis of prostaglandins [42, 44].

Mitochondria is the principal site for energy production at cellular level, in the form of adenosine triphosphate (ATP), a process in which peroxides and free radicals such as reactive oxygen and nitrogen species (ROS/RNS) also result as products [43]. Glucose is a key element for cellular energy production. At mitochondrial level, during the glycolysis process, glucose brakes down and generates two molecules of pyruvate, which is converted into acetyl coenzyme A (acetyl-CoA). Acetyl-CoA enters the Krebs cycle reactions and generates nicotinamide adenine dinucleotide (NAD^+^), flavin adenine dinucleotide (FAD), and guanosine diphosphate (GDP) [45, 46].

During the Krebs cycle reactions, NAD^+^ and FAD are reduced to NADH and to FADH_2_, these two being key elements in the electron transporting chain, as well as in the production of ATP via the oxidative phosphorylation process [47]. Furthermore, under the action of succinyl-CoA synthetase GDP, inorganic phosphate and succinyl-CoA undergo a phosphorylation reaction that leads to GTP, coenzyme A, and succinate. Afterwards, succinate and ubiquinone (Q) suffer an oxidation reaction mediated by succinate dehydrogenase that leads to fumarate and ubiquinol (QH_2_). The last reactions of the cycle include malate production from fumarate (mediated by fumarase), oxaloacetate from malate, and the synthesis of citrate and coenzyme A from oxaloacetate and acetyl-CoA. ATP can also be produced from GTP under the action of nucleoside-diphosphate kinase [48].

Each molecule of glucose that enters the Krebs cycle produces at the end of the cycle two GTP, six NADH, two ubiquinol, and four molecules of carbon dioxide. It is reported that each molecule of NADH that results during the Krebs cycle correlates with a production of 2.5 molecules of ATP, whereas FADH_2_ produces only 1.5 ATP molecules. The total amount of ATP obtained from one molecule of glucose that undergoes glycolysis and oxidative phosphorylation during the Krebs cycle is up to 38 molecules [48, 49]. As mentioned previously, we must underline the importance of NADH and FADH_2_ due to their role in the electron transporting chain. During the Krebs cycle reactions, a small number of electrons (estimated at 1-3% of all electrons) escape the respiratory transport chain towards oxygen, thus generating oxygen-derived free radicals [41].

The electron transport chain across the inner mitochondrial membrane (IMM) in order to produce ATP is mediated by a series of mitochondrial protein complexes that allow the passage of electrons from donors to more electronegative accepting molecules; this process repeats itself until the electrons reach their final acceptor which is oxygen. The energy released during the electron transport is used to transfer protons across the IMM [50]. Therefore, the electron transport chain is coupled with the transport of protons from the mitochondrial matrix across the IMM. This process leads to a proton gradient that has an essential role in the mitochondrial membrane potential [45, 51].

This entire process of electron transport is mediated by four protein complexes that are strongly attached to the IMM: NADH-ubiquinone oxidoreductase (complex I), succinate dehydrogenase also known as succinate-ubiquinone reductase or succinate-coenzyme Q reductase (complex II), cytochrome bc1 complex (complex III), and cytochrome c oxidase (complex IV) [52, 53]. Another important aspect that facilitates the electrons transport is the fact that these complexes are linked with water and lipid soluble electron carriers. Out of these four complexes, only three of them act as proton pumps, being able to transfer protons across the IMM: complexes I, III, and IV [45].

Complex I has a major role in the electron transporting chain, being the site where electrons enter the respiratory chain. At this level, NADH suffers an oxidation reaction that leads to NAD^+^ and to the release of two electrons. These electrons are taken by a lipid soluble electron carrier known as ubiquinone (coenzyme Q—CoQ) that undergoes a reduction reaction becoming ubiquinol (UQH_2_), and it passes across the IMM. This process is coupled with the transfer of four protons which generate a proton gradient [45]. It is estimated that the energy released during these transfers accounts up to 40% of the energy necessary for ATP synthesis, as well as for the transport of other important factors to and from the mitochondria. Complex I is one of the first sites of electrons leakage, leading to oxygen-derived free radicals, especially to superoxide radical. It is worth mentioning that ubiquinone also receives electrons from complex II [54–56].

Complex II also transports electrons, these electrons being delivered from FADH_2_ to ubiquinone in parallel with the complex I electron mediated transfer, but in contrast to complex I proton transfer capabilities, the type II complex does not transfer protons to the intermembrane space. This complex mediates the oxidation reaction of succinate that leads to fumarate and also the conversion of ubiquinone into ubiquinol [45]. During the reactions that take place throughout the electrons transfer process, ubiquinone suffers a reduction reaction that leads to semiquinone. This unstable intermediate of ubiquinone may also lead to superoxide radical production [57–59].

Complex III, known as cytochrome bc1, receives electrons from both complexes I and II via coenzyme Q (ubiquinone) and transfers them to cytochrome c, this process being accompanied by proton transport. Similar to complex I, cytochrome bc1 is also involved in oxygen-derived free radical production, due to the fact that electrons may leak towards oxygen during the electron transfer reactions. The amounts of ROS produced by complex III are significantly inferior compared to those produced by complex I [60]. Several researchers have emitted the hypothesis that complex III-associated ROS may have positive effects in terms of cellular adaption to ischemic events, which suggests a beneficial cardioprotective outcome, as well as in apoptosis [61, 62].

Complex IV (cytochrome c oxidase) receives electrons from cytochrome c and transfers them to oxygen, which is the last electron acceptor of this transporting chain, thus reducing it to water. Complex IV as a component of the proton gradient, in parallel with the electron transfer process, it also transports four protons across the inner mitochondrial membrane therefore maintaining a normal transmembrane proton gradient [56]. This proton gradient is essential for ATP production, because it permits the ATP synthase to use the protons in order to obtain ATP from ADP and inorganic phosphate [45, 63].

Another site for ROS production is represented by the peroxisomes. These oxidative organelles found in the cytoplasm of eukaryotic cells are involved in several important metabolic reactions: the catabolism of fatty acids, long amino acids, bile acid-derived intermediates, polyamines, the plasmalogens synthesis, and ROS reduction [41, 64]. Enzymes found in peroxisomes use molecular oxygen as a cosubstrate during metabolic reactions, remove hydrogen from different molecules involved in these reactions, and form oxygen-derived free radicals (only H_2_O_2_). H_2_O_2_ is used by catalase (another peroxisomal enzyme) during the oxidative reactions of several agents (alcohol, formic acid, and phenols), reactions during which H_2_O_2_ is transformed into water and O_2_, thus reducing its accumulation as well as its negative impact [65].

Free radicals are characterized by the fact that they have one or more unpaired electrons, this characteristic allowing them to interact without any major difficulties with other molecules, thus leading to chain reactions that can prove to be dangerous to normal cellular homeostasis [41, 50].

The most important types of free radicals are those derived from oxygen (superoxide anion radical O·_2_^−^, hydrogen peroxide H_2_O_2_, and hydroxyl radical ·OH). Superoxide anion radical is considered the principal ROS, being responsible for the production of secondary ROS (H_2_O_2_ and ·OH). O·_2_^−^ results, as we have previously described, during the mitochondrial respiratory chain reactions and under the action of NADPH (dinucleotide phosphate) oxidase [43]. Another type of free radicals that could arise during the respiratory chain reactions (especially in hypoxic conditions) is nitrogen-derived free radicals such as nitric oxide that can further lead to other free radicals (nitrite (NO_2_^−^), peroxynitrite (ONOO^−^), hypochlorous acid, aldehydes, and 4-hydroxynonenal) [42, 66].

Molecular oxygen is characterized by the fact that it can easily accept an electron leaked during respiratory chain reactions, thus leading to superoxide anion radical. Under the action of an enzyme known as superoxide dismutase (SOD), O·_2_^−^ suffers a dismutation reaction that leads to H_2_O_2_ production [43, 67]. Furthermore, the interaction between hydrogen peroxide and superoxide anion radical will lead to hydroxyl radical. Another mechanism responsible for the production of ·OH is the cleavage of H_2_O_2_, reaction mediated by Cu^2+^ or by Fe^2+^ [41, 68].

Several reports have described other possible sources for these previously mentioned ROS, excluding the mitochondrial respiratory chain: alterations of the mitochondrial membrane potential and two mitochondrial enzymes, one located in the space between the mitochondrial membranes and another one attached to the outer mitochondrial membrane (p66Shc, respectively, monoamine oxidase), as well as several enzymes involved in the Krebs cycle (pyruvate dehydrogenase, *α*-ketoglutarate dehydrogenase) [57, 69–71].

There are other free radicals that result from oxygen, like peroxyl radicals (ROO·) (hydroperoxyl and perhydroxyl radicals). These peroxyl radicals play a major role in lipid peroxidation via fatty acid peroxide-dependent and fatty acid peroxide-independent pathways [41, 72].

In order to face the negative impact of the OS, cells are equipped with several enzymes that interfere with the action of these previous described free radicals:

- Superoxide dismutases (three isoforms have been identified—SOD1-3)—use superoxide anion and convert it into O_2_ and H_2_O_2_
- Catalase—scavenges H_2_O_2_, transforming it into O_2_ and H_2_O
- Glutathione peroxidases, glutathione reductase, glutathione S transferase, glutathione disulfide, and nonenzyme glutathione—involved in H_2_O_2_ neutralization
- Thioredoxins 1 and 2, peroxiredoxins, lipoic acid, L-arginine, and coenzyme Q10 [41, 42].

Mitochondria are the principal site for ROS production, but it is at the same time an important target of OS and ROS. OS modifies the mitochondrial membrane permeability, reducing it, as well as the mitochondrial membrane potential [36, 44]. This will lead to a series of reactions that consist in the release of cytochrome C and B-cell lymphoma into the cytosol, followed by the assembly of the apoptosome (process mediated by apoptotic activating factor I) and the activation of the capsase-9. Furthermore, the latter activates caspase-3 thus initiating apoptosis. Another important trigger of the caspase-induced apoptosis is 4-hydroxynonenal, a metabolite that results from the lipid peroxidation process [44, 73, 74]. ROS induce mitochondrial DNA damage via oxidative reactions, and they also affect mitochondrial lipids and proteins [57]. Molecular-driven research has reached the conclusion that nuclear DNA has a significantly lower risk of ROS-associated damage compared to mitochondrial DNA, this being explained by the fact that nuclear DNA has a well-developed protection mechanism represented by histones and also because of the nucleotide excision repair mechanism, which is less represented in the mitochondrial DNA [75, 76].

## 4. Molecular Events Related to Oxidative Stress in POP

Transforming growth factor-*β*1 (TGF-*β*1) is a well-known cytokine involved in numerous fibrotic processes. Sustained mechanical stress leads to the release of TGF-*β*1. At cellular level, TGF-*β*1 binds to its receptor TR*β*II (TGF-*β* receptor II), this process being mediated by the Smad transcription factors (mothers against decapentaplegic homolog). These transcription factors included in the Smad family are essential for the TGF-*β*1 signaling pathway, because they mediate the signal transduction from TR*β*II to the nucleus [77, 78]. Once activated, TGF-*β*1 has an important role in fibrogenesis, as well as in the extracellular matrix remodeling process, controlling the deposition of important ECM components, as well as the expression of MMPs and TIMPs. TGF-*β*1 stimulates fibroblasts differentiation, collagen synthesis (especially type I and II collagens by myofibroblasts), and it also decreases the collagen degradation process, by blocking the action of MMPs and also by increasing the expression of TIMPs [14, 79]. TGF-*β*1 signaling seems to be also mediated by other pathways, different from the Smad family: the phosphoinosite-3-kinase/Akt pathway [80].

**Figure 1:**
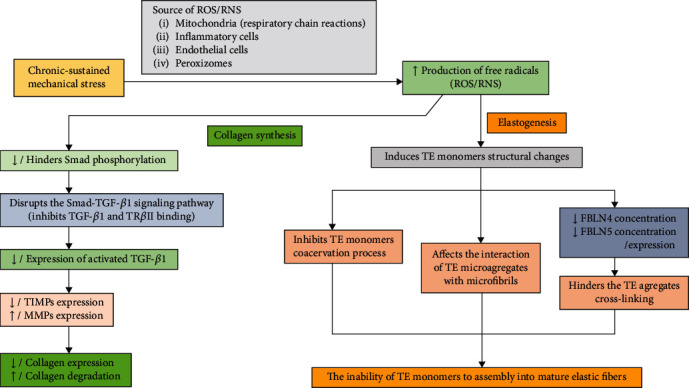
The impact of OS upon collagen synthesis and elastogenesis.

Considering the fact that the ECM has an important role in POP pathogenesis, a study from 2017 has focused on the effects of excessive mechanical stress upon human uterosacral ligament fibroblasts (hUSLFs), as well as on their overexposure to H_2_O_2_. The tissue samples were taken from 15 patients who have undergone vaginal hysterectomy for nonneoplastic pathologies and have been subjected to mechanical stress with different intensities and were also exposed to H_2_O_2_ at different concentrations. This has led to a significant decrease of the mitochondrial DNA level, as well as of ECM protein expression. It was noticed that elastin expression and several collagen precursors (COL 1A1 and COL 3A1) were inhibited, and lower H_2_O_2_ concentrations stimulated the expression of extracellular matrix proteins. This was also noticed when smaller forces were applied to the hUSLFs tissue samples. Another important observation is the fact the MMP levels had increased, whereas the TIMP expression had suffered a decline, thus favoring ECM degradation. The authors have also investigated the impact of H_2_O_2_ overexposure and sustained mechanical stress upon the Smad/TGF-*β*1 pathway. It was found that the expression of phosphorylated Smad2 had declined and also the levels of activated TGF-*β*1. Therefore, the authors have concluded that excessive mechanical stress and H_2_O_2_ overexposure interfere with Smad/TGF-*β*1 pathway, significantly affecting the ECM remodeling process and favoring POP [37] (Figure 1).

Another study investigated the role of TGF-*β*1 in POP pathogenesis. 60 patients who have undergone hysterectomy for nonmalignant pathologies have been divided in two groups, as follows: a study group (30 patients) that included only patients with POP (from stage II to stage IV) and a control group without POP. Tissue samples have been taken from the uterosacral ligaments during surgery, in order to evaluate the expression of collagen, elastin, MMP 2, MMP 9, TIMP 2, TGF-*β*1, and Smad. The purpose of these investigations was to establish the connection between OS inducing factors and POP pathogenesis. The authors have noticed that the collagen and elastin from the POP samples have presented important structural changes and also a significant decline of their expression compared to the control group (lower levels of COL 1A1, COL 3A1, and elastin in the study group). Differences between the study group MMPs/TIMPs expression and the control group were also found. Therefore, the POP group presented significantly higher levels of MMP 2 and MMP 9 compared to the non-POP group, as well as a lower TIMP 2 expression compared to control group, where the TIMP 2 levels were higher. In regards to the TGF-*β*1 expression, the study group associated lower expression rates compared to the non-POP patients. Further analysis that aimed at assessing the correlation between POP severity and TGF-*β*1 levels has revealed that as the POP severity increased, the TGF-*β*1 expression decreased. Another important hypothesis over which the authors have focused their attention is the ability of TGF-*β*1 to minimize the mechanical stress-induced ECM degradation. In order to investigate this theory, the authors have treated fibroblast originating from the uterosacral ligaments with various concentrations of TGF-*β*1 (0, 5, or 10 ng/ml) for 4 hours. Afterwards, the fibroblasts have been subjected to cyclical mechanical stress (5,333-*με*) for approximately 24 hours. It was found that TGF-*β*1-treated hUSLFs associated a significantly higher viability rated compared to those without TGF-*β*1 treatment. When analyzing the cell population without TGF-*β*1 treatment, the authors have noticed that the COL 1A1, COL 3A1, and elastin rates were significantly lower compared to the TGF-*β*1 treatment group, as well as higher expressions of MMP 2/MMP 9 and a significantly lower expression of TIMP 2. Therefore, they have concluded that treatment with TGF-*β*1 could significantly reduce mechanical stress-associated collagen and elastin degradation. Another important mechanism that was investigated in this study was the TGF-*β*1/Smad signaling pathway. The authors have found that hUSLFs subjected to mechanical stress have associated a significantly lower Smad2 and 3 phosphorylation rate, whereas in the TGF-*β*1-treated group, it was notice that treatment with TGF-*β*1 can significantly improve the Smad3 phosphorylation rate. TGF-*β*1 pretreating did not improve the Smad2 phosphorylation rate. The published results sustain the already existing data regarding the fact that mechanical stress induces molecular changes at the level of the pelvic-supportive structures, changes that can lead to POP. Another important conclusion of this study was that TGF-*β*1 treatment could significantly reduce the risk of these molecular changes [14].

The impact of OS because of mechanical stress has been evaluated in several clinical studies. Hong and his colleagues have assessed the impact of mechanical stress on human parametrial ligament fibroblasts (HPLFs) in terms of oxidative damage that could favor POP pathogenesis. Tissue samples have been taken from ten patients who have undergone hysterectomy. The surgery was performed for nonmalignant pathologies. The patients included in this study did not present any collagen disorders or any other pathologies that could be associated with OS. The tissue samples have been subjected to mechanical forces of variable intensities for four hours (1,333, 2,666, or 5,333-*μ* strains). The researchers have noticed that the mechanical forces have led to cellular morphology alterations, such as cellular atrophy, and to the weakening of the cellular junctions. These cells have also undergone a deformation process, becoming round, despite the fact that initially they were long spindle cells. In terms of ROS production under the action of mechanical stress, the authors have reported that low mechanical strains were associated with a small increase of ROS levels, these levels significantly increasing with the intensity of the mechanical stress (especially when a 5,333-*μ* mechanical strain was applied). Another important aspect that this study has analyzed was the correlation between the degree of mechanical stress and the apoptotic rate of HPLFs. A lower mechanical strain of 1,333-*μ* did not increase the cellular apoptotic rate, whereas as higher forces like 2,666 and 5,333-*μ* strains have led to higher apoptotic rates. The cells that have been subjected to 5,333-*μ* strains have suffered a fragmentation process and only half of them were nonnecrotic cells, but out of these nonnecrotic cells, up to 50% of them were apoptotic. In the group of cells that have been subjected to lower mechanical forces, the cellular fragmentation process was not so pronounced, so the percentage of the nonnecrotic cells was significantly higher, being up to 95% in the 2,666-*μ* strain group. The authors have explained that this high percentage of nonnecrotic cells could be explained by the short period of time the cells were exposed to those forces [36].

In a study published in 2013, Kim and his collaborators have investigated the role of OS and mitochondrial apoptosis in the pelvic organ prolapse pathogenesis. The authors have assessed the presence of several biomarkers in tissue fragments that have been taken from the uterosacral ligaments: 8-hydroxy-2-deoxyguanosine (8-OHdG; biomarker of DNA oxidative damage) and 4-hydroxy-2-nonenal (4-HNE; biomarker of lipid peroxidation). The study group included 26 advanced POP patients that have undergone hysterectomy and sacrocolpopexy, whereas the control group included 29 patients with maximum stage I POP, these patients undergoing surgery for different benign pathologies. The tissue fragments have been divided in two groups, one for Western blot analysis, and the other one for immunohistochemistry and TUNEL (terminal deoxynucleotidyltransferase-mediated deoxyuridinetriphosphate nick end labeling) assay. The markers used for the evaluation of mitochondrial apoptosis were cytochrome c, cleaved capsase-3, and capsase-9. The immunohistochemical investigation of the extracted tissue fragments has revealed the presence of the OS markers in all the tissues samples, including in the fragments that have been taken from the control group. In spite of these findings, the expression of 8-OHdG and 4-HNE was significantly higher in the study group. Therefore, approximately 40.39% ± 11.08% of the analyzed tissue samples from the study group were positive for 8-OHdG and 46.04% ± 10.86% for 4-HNE, whereas in the control group, the percentages were 18.27% ± 7.19% and 18.61% ± 8.11%, respectively [44].

All of the biomarkers that have been used to evaluate the mitochondrial apoptosis have also associated significantly higher expressions in the study group compared to those in the control group, regardless of the used investigation method. The authors have reported that the increased levels of 8-OHdG and 4-HNE have also correlated with the mitochondrial apoptosis biomarkers. In terms of POP degree, the expression of 8-OHdG, 4-HNE, and cytochrome c was significantly higher in the study group samples compared to the control group, regardless of the POP stage, but the expression of caspase-3 was found to be high only in the tissue samples that came from high-grade POP patients [44].

A similar study that has investigated the role of OS in terms of collagen-associated disorders that could lead to pelvic organ prolapse was published by Liu and his colleagues. The study included 40 patients who have undergone hysterectomy, these patients being divided in two groups (study group versus control group). The study group included only POP patients (from stage II to stage IV POP), whereas the patients included in the control group did not associate POP, the surgery being done for benign pathologies. Tissue samples taken from the parametrial ligaments (uterosacral and cardinal ligaments) have been subjected to H_2_O_2_ exposure (concentrations 0.1, 0.2, 0.4, 0.6, 0.8, and 1 mM) for periods of time that ranged between four and 24 hours. OS damage was evaluated using immunohistochemistry in order to detect the 8-OHdG and 4-HNE expression, and it was found that the expression of these markers was significantly higher in the study group compared to non-POP patients. In terms of H_2_O_2_-associated toxicity, the authors have noticed that this was influenced by H_2_O_2_ concentrations, as well as by the period of time in which the tissue samples have been exposed to H_2_O_2_. The results found after a 24 hours exposure have revealed that the percentage of cell viability was significantly lower compared to a 4-hour exposure. A H_2_O_2_ concentration of 0.1 mM was associated with negligible cytotoxicity, whereas in the case of higher concentrations, the cytotoxic effect was markedly increased (0.2 mM associated a moderate to severe cytotoxic effect, while concentrations greater than 0.4 mM associated severe cytotoxicity). A similar correlation was also found in terms of cellular apoptosis and H_2_O_2_ concentration. The impact of H_2_O_2_ on the collagen metabolism was evaluated based on COL 1A1, MMP 2, TIMP 1, and TGF-*β*1 expression. The results obtained by the authors have revealed that as the H_2_O_2_ concentration grew, the expression of COL 1A1, TIMP 1, and TGF-*β*1 had registered a steady decline. In terms of MMP 2, the results have revealed that its expression had increased in parallel with the H_2_O_2_ concentration. The authors have also mentioned that lower concentrations of H_2_O_2_ had stimulated COL 1A1 synthesis, thus suggesting an anabolic effect of low-dose H_2_O_2_, whereas higher concentrations have had the opposite effect, stimulating the COL 1A1 catabolism. Considering these results, the authors have concluded that OS interferes in collagen homeostasis and metabolism, leading to molecular changes that may play a key role in POP pathogenesis [38].

The elastogenesis process is another important key point that can be affected by OS, which generates a series of molecular changes that significantly contributes to the POP pathogenesis. The assembly of the elastic fibers is a complex process that involves several steps: elastogenic cells secrete TE; TE molecules form microaggregates (coacervation process); TE monomers form larger aggregates (cross-linking process—mediated by FBLNs 4 and 5), which are deposited on microfibrils; and LOX-mediated elastic fiber assembly of the aligned and cross-linked TE aggregates [26, 34].

The implications of OS upon elastogenesis, as well as the exposure of TE to reactive nitrogen species (RNS), have raised the interest of several researchers that aimed at finding correlations between these processes and POP pathogenesis. Considering the fact that elastogenesis is usually initiated in an inflammatory context, Akhtar and his colleagues have exposed TE molecules to ONOO^−^ and HOCl (both substances being released by macrophages and monocytes during inflammatory processes). The authors have identified several elastogenesis key points that could be affected as a result of OS. TE monomers exposed to ONOO^−^ and HOCl have developed structural changes that significantly interfered with their assembly into elastic fibers. A drop of up to ten degrees in the coacervation temperature was noticed in the ROS/RNS TE group compared with the normal TE group, this fact significantly influencing the elastic fiber assembly process. The cross-linking phase was also affected, this process being evaluated based on desmosine levels. It was noticed that TE monomers exposed to ONOO^−^ and HOCl have associated significant lower desmosine rates compared with the nonexposed TE molecules. Further investigations have focused on the relation between FBLN 4/FBLN 5 and the cross-linking process. Both FBLN 4 and FBLN 5 have registered a significant decline in terms of their binding capabilities to ROS/RNS-modified TE molecules, thus suggesting that ROS/RNS agents affect normal elastogenesis (Figure 1). In this study, the authors have reported that the interaction rate between FBLN 4 and ONOO^−^/HOCl-exposed TE had registered a decline with approximately 35% (for both agents), whereas in the case of FBLN 5, the decline rate was even higher (50%). Another important step of the elastic fibers assembly process is the interaction between TE and microfibrils. The authors have also investigated this process, analyzing the interactions between ROS/RNS-exposed TE molecules and FBN 2/MAGP 1, these two being essential components of the microfibrils. They have reported that the interaction between FBN 2 and ONOO^−^/HOCl-exposed TE had decreased with up to 55% and 25%, respectively. No significant differences have been reported regarding the manner in which MAGP 1 has interacted with TE exposed to ONOO^−^/HOCl or with normal TE molecules [34]. This study has demonstrated that both ROS and RNS agents can significantly disturb normal elastogenesis, interfering with its numerous key points and altering the integrity of the pelvic-supportive structures (Figure 1).

## 5. Conclusions

OS is an important factor involved in pelvic organ prolapse pathogenesis, being responsible for numerous molecular changes at the level of pelvic-supportive structures that weaken the integrity and the resistance of pelvic muscles, fascias, and ligaments, thus favoring the development of this pathology.

Sustained mechanical stress is proven to be a key factor in the appearance of pelvic organ prolapse, correlating with increased levels of free radical production and mitochondrial-induced fibroblasts apoptosis, the rate of cellular apoptosis, depending on the intensity of the mechanical stress, and the period of time the mechanical stress is applied.

Mitochondria, besides being one of the main sources of free radicals production, are also the site of their action. OS via ROS/RNS hinders the normal MMPs/TIMPs balance, favoring MMP overexpression and activity in the detriment of TIMP activity, significantly altering the collagen synthesis process. ROS/RNS interfere with the Smad/TGF-*β*1 pathway, this being essential for collagen homeostasis, by decreasing the Smad phosphorylation rate and furthermore the levels of activated TGF-*β*1. Current studies underline the potential benefits of TGF-*β*1 treatment in terms of reducing the collagen and elastin degradation rate.

Another important process over which the OS manifests its negative effects is elastogenesis, ROS/RNS acting at the level of its numerous key point reactions, significantly damaging the elastic fiber assembly process and therefore favoring the prolapse of the pelvic organs.
